# Enhancing the structural stability of P29-targeted monoclonal antibodies via β-hydroxybutyrylation modification improves their therapeutic performance in alveolar echinococcosis

**DOI:** 10.3389/fcimb.2025.1716047

**Published:** 2026-01-05

**Authors:** Shiqin Yuan, Tao Li, Lei Niu, Yazhou Zhu, Lang Wu, Wei Zhao, Ming Li, Zihua Li

**Affiliations:** 1 School of Basic Medicine, Ningxia Medical University, Yinchuan, Ningxia, China; 2 Key Laboratory of Prevention and Control of Common Infectious Disease, Ningxia Medical University, Yinchuan, Ningxia, China; 3 Ningxia Eye Hospital, People’s Hospital of Ningxia Hui Autonomous Region, Ningxia Medical University, Yinchuan, Ningxia, China; 4 Department of Hepatobiliary Surgery, General Hospital of Ningxia Medical University, Yinchuan, Ningxia, China; 5 Department of Hepatobiliary Surgery, People’s Hospital of Ningxia Hui Autonomous Region, Ningxia Medical University, Yinchuan, Ningxia, China

**Keywords:** alveolar echinococcosis, antibody, antiparasitic therapy, protein engineering, β-hydroxybutyrylation

## Abstract

**Background:**

Alveolar echinococcosis (AE), a severe parasitic infection often likened to "parasitic cancer", still lacks effective treatments. Although our earlier ork on the P29 monoclonal antibody (P29 mAb) against the parasite-derived P29 antigen showed potential, its efficacy remained limited, prompting the need for improved biologic agents.

**Methods:**

We applied β-hydroxybutyrylation (Kbhb) modification to engineer an enhanced antibody, P29 mAb^Kbhb^, and comprehensively evaluated its properties using surface plasmon resonance, protease resistance assays, pharmacokinetic studies in C57BL/6 mice, and histopathological analysis of alveolar hydatid cysts.

**Results:**

The modified antibody retained high antigen-binding affinity (KD = 343 pM) and exhibited markedly increased resistance to proteolytic degradation, with a 1.75-fold improvement in serum persistence after 5 weeks. Furthermore, in a murine model of AE, P29 mAb^Kbhb^ significantly inhibited protoscolex regeneration and induced apoptosis of cyst wall cells relative to the unmodified antibody.

**Conclusion:**

Our results establish a novel connection between protein engineering and antiparasitic therapy, illustrating that Kbhb modification not only augments the efficacy of anti-AE antibodies but also offers a versatile strategy for enhancing antibody stability and half-life. This offers a potential strategy for developing new treatments against neglected zoonotic diseases via tailored post-translational modifications.

## Introduction

Hydatid disease, also known as Echinococcosis, is a zoonotic parasitic disease caused by the parasitic infestation of *Echinococcus larvae* on intermediate hosts such as rodents or humans, which seriously endangers human health and affects socio-economic development (Kern, 2003; McManus et al., 2003; Mehta et al., 2016; Shafiei et al., 2016; Wen et al., 2019). Human echinococcosis is mainly divided into two types: cystic echinococcosis (CE) and AE. It is predominantly prevalent in Europe, Eastern and central Asia, Northern Africa, South America, and parts of Oceania, especially in countries dominated by animal husbandry, such as New Zealand, Australia, Argentina, Canada, and China (Deplazes et al., 2017; Pianciola et al., 2017; Kotwa et al., 2019; Bardsley et al., 2022). According to the data from the World Health Organization, there are approximately 18,200 new cases of AE worldwide each year. The mortality rate of untreated AE patients is as high as 90% in 10–15 years (Kern et al., 2017). What’s more, because of the difficulty of surgery, AE is more dependent on drug therapy. However, the reported therapeutic efficacy of AE remains unsatisfactory, with a treatment period for this condition up to 43 years with a recurrence rate of 37% after 1 to 6 years of follow-up (Torgerson et al., 2018). Albendazole, a first-line anti-echinococcosis agent, can cause significant adverse effects during prolonged administration, especially hepatorenal toxicity cannot be ignored (Dragutinović et al., 2022).

In recent years, with the rapid development of immunology, immunotherapy has become a novel treatment approach for various diseases including tumors, autoimmune diseases, metabolic diseases, and infectious diseases. Among these, monoclonal antibodies have shown particularly outstanding effects in the field of disease targeted therapy (Schmid and Neri, 2019; Lu et al., 2020; Promsote et al., 2020; Briani and Visentin, 2022; Chavda et al., 2022; Keam, 2023; Paul et al., 2023; Swain et al., 2023; Zhang et al., 2023). However, compared to tumors and other pathogenic conditions, research on antibody therapy for echinococcosis is still in its infancy. As targeted drugs, the specific antigen selection is crucial for the therapeutic effect of antibody drugs. Based on previous research on molecular vaccines, our group have identified a P29 antigen that provides good immune protection against echinococcosis. This molecule has been proven to offer over 90% immune protection in experiments with mice and sheep (Shi et al., 2009; Wang et al., 2016). Additionally, we found that the level of anti-P29 IgG in the serum of patients with active echinococcosis is significantly elevated, suggesting that the immune protection provided by P29 may be partly due to the specific antibodies it induces (Tao et al., 2022). Therefore, we next screened a monoclonal antibody against P29 (P29 mAb, 4G10F4) with high antibody titer using traditional hybridoma cell fusion technology. Both *in vitro* studies on the inhibition of alveolar hydatid cyst wall cells and *in vivo* experiments on alveolar hydatid cysts have demonstrated that this antibody exhibits certain therapeutic effects; however, its efficacy remains below anticipated levels (Zhang et al., 2024). Thus, how to enhance the therapeutic effect of the P29 mAb is the focus of this study.

3-Hydroxybutyric acid is an endogenous small molecule in the human body that promotes glucagon secretion due to decreased blood glucose levels during exercise, hunger, or glucose metabolism disorders. It can provide energy for various cellular activities or participate in numerous physiological regulatory processes as a signaling molecule (Li et al., 2021; Yao et al., 2021; Zhang et al., 2021). Recent studies have found that 3-hydroxybutyrate also plays a significant role in epigenetic regulation. The carboxyl group of 3-hydroxybutyric acid forms a covalent bond with the free amino group of lysine on histones, resulting in a new type of post-translational modification (PTM) known as protein kbhb. This modification is widely present in yeast, fruit flies, mice, and human cells, regulating the expression of related target genes (Xie et al., 2016; Chen et al., 2017; Zhang et al., 2020; Li et al., 2022). We previously analyzed the total protein in the spleen of mice through proteomics and first discovered the kbhb modification of antibodies, which can enhance the stability of antibodies against proteases (Li et al., 2022). But the function and physiological significance of kbhb modification on antibody remain unclear. Therefore, we hypothesized that combining the anti-cystic effect of P29 mAb with the stabilizing effects of kbhb modification on the antibody would synergistically enhance the therapeutic efficacy of P29 mAb in treating AE. In this study, we systematically investigated the impact of kbhb modification on the structural-functional characteristics of P29 mAb, as well as the therapeutic potential of the modified antibodies in a AE model, aiming to provide new strategies for antibody therapy in hydatid disease.

## Methods

### Animal experiment

Six-week-old female C57BL/6 mice were procured from the accredited Experimental Animal Center of Ningxia Medical University and maintained under strict specific pathogen-free (SPF) conditions. The animals were housed in temperature-controlled (23 ± 1 °C) individually ventilated cages with a standardized 12-hour light/dark cycle. Standard rodent chow and sterile water were available ad libitum. For sample collection, experimental animals were anesthetized by intraperitoneal injection of tribromoethanol (125–150 mg/kg). After reaching a surgical plane of anesthesia, blood samples were collected. Euthanasia was then immediately performed via an overdose of tribromoethanol (additional 150–200 mg/kg), confirmed by cervical dislocation. Tissue samples (including liver and spleen) were collected following verification of death. All experimental protocols were designed and conducted in strict accordance with the National Guide for the Care and Use of Laboratory Animals, and were approved by the Experimental Animal Ethics Committee of Ningxia Medical University (Approval No. IACUC-2025102), ensuring full compliance with ethical and welfare standards.

### Kbhb modification of P29 mAb and quantification

The unmodified P29 mAb was synthesized by Zoonbio Biotechnology Company (Shanghai, China). Half of the preparation were used as a control, while the remaining portion were diluted to 1 µg/µL in PBS buffer. Chemical modification was initiated by adding 10 mM 1-(3-dimethylaminopropyl)-3-ethylcarbodiimide hydrochloride (EDC) and 10 mM 3-hydroxybutyric acid to the antibody solution, followed by gentle agitation for 2 h at room temperature (RT). To remove residual unreacted compounds, the mixture was purified using 30-kDa molecular weight cutoff (MWCO) centrifugal filters with three PBS washes (10 min, 4,000 rpm each). The retained fraction containing β-hydroxybutyrylated P29 mAb (P29 mAb^kbhb^) was concentrated and collected. Both modified and unmodified antibodies were quantified via BCA assay (KeyGen, China, Cat. No. KGB2101-500). For statistical reliability, three independent experimental replicates were performed, each with triplicate measurements, and the respective mean concentrations were calculated. Based on prior optimization data for murine AE treatment (Zhang et al., 2024), the final concentrations of both P29 mAb and P29 mAb^kbhb^ were adjusted to an equivalent level of 0.5 µg/µL for subsequent experiments. The success of kbhb modification was confirmed by Western blotting analysis using an anti-kbhb specific antibody (PTM BIO, PTM-1201RM), with unmodified P29 mAb serving as the negative control.

### ELISA

The 96-well plates were coated with recombinant *Echinococcus multilocularis* P29 antigen (rEm.P29) at three concentrations (0.1, 1, and 10 μg/mL) in coating buffer (84.87 mM NaHCO_3_, 15 mM Na_2_CO_3_, pH 9.5), followed by overnight incubation at 4 °C. After five 1-minute washes with PBST, nonspecific binding sites were blocked with 5% (w/v) skim milk prepared in PBST (200 μL/well, 37 °C, 1 h). Primary antibody dilutions (1:400 to 1:400,000) were then added at 100 μL/well and incubated at 37 °C for 2 h. Following another washing cycle, HRP-conjugated secondary antibody (1:10,000 in blocking buffer) was added and incubated at 37 °C for 1 h protected from light. After seven final PBST washes, the reaction was developed with TMB substrate (PR1210, Solarbio, China) at 100 μL/well for 5 min at RT in the dark, and stopped with 50 μL of stop solution (C1058, Solarbio, China) before measuring absorbance at 450 nm using a microplate reader (Thermo Fisher). All washing steps were performed using PBST (0.15% Tween-20) with 1-minute incubation per wash.

### Isolation and viability detection of protoscolex in alveolar hydatid

*Echinococcus multilocularis* protoscoleces (PSCs) possess the primordial scolex structure with the potential to mature into adult worms and serve as the infectious component within alveolar hydatid cyst. The PSCs were obtained from intraperitoneal alveolar hydatid cysts of mice maintained in our laboratory. The right lower abdominal cavity of 6 weeks old mice was disinfected with iodine and injected with 200 μL of PSCs suspension (10 PSCs per μL). After 3–5 months of growth, the cysts were extracted from euthanized mice, placed in a 10-cm culture dish, and rinsed three times with sterile PBS. Subsequently, an ophthalmic scissor was used to finely chop the cyst tissue blocks until a homogenate was formed. The minced tissue homogenate was transferred to a metal filter screen (0.18 mm pore diameter) and gently ground with the plunger of a 5 mL syringe while rinsing with PBS, allowing PSCs to separate from the cyst tissue and flow into a 20 cm glass culture dish. The filtrate containing the PSCs was collected into a 50 mL sterile centrifuge tube and filtered through a screen (0.15 mm pore diameter) to remove smaller tissue fragments. After natural precipitation of PSCs, the supernatant was discarded, and the PSCs pellet was washed three times with sterile PBS. Next, a 10-fold volume of 1% pepsin solution (pH 2.0) was added to the PSCs pellet, mixed thoroughly, and incubated at 37°C for 30 min. The suspension was gently inverted 3-5 times every 5 min to ensure complete digestion of adhered tissue fragments. After allowing the PSCs to settle for 5 min, the supernatant was discarded and the pellet was washed three times with PBS. The PSCs precipitate was resuspended in PBS, and three 10 μL aliquots of suspension were placed on glass slides. Each aliquot was stained with 1 μL of eosin solution (D019-1-2, Njjcbio, Nanjing, China). Viable PSCs exclude the dye and remain unstained, whereas non-viable PSCs with compromised membranes take up the dye and turn red. Therefore, the morphology of PSCs was observed under an optical microscope (Nikon, Japan). The total number of PSCs and the number of eosin-stained PSCs in each 10 μL aliquot were counted. The viability of isolated PSCs was calculated as follows: % viability = [(total number of PSCs - number of stained PSCs)/total number of PSCs]×100%. Finally, the PSCs suspension was replaced with complete culture medium (90% DMEM, 10% FBS, 1% penicillin-streptomycin, and 10 μg/mL ciprofloxacin) and cultured aseptically in a 10 cm culture dish for subsequent use.

### Western blotting

A total of 20-50 μg of r*Em*.P29 protein was mixed with 1×SDS loading buffer (P1040, Solabao, China), and then boiled for 10 min for denaturation. The denatured protein samples were then separated by 10% sodium dodecyl sulfate-polyacrylamide gel electrophoresis (SDS-PAGE) at a constant voltage of 100 V. Next, the separated proteins were transferred from gel to polyvinylidene fluoride (PVDF) (Millipore, Billerica, MA, USA) at a constant current of 260 mA for 2 h at 4°C. To prevent nonspecific binding, the PVDF membrane was blocked with 5% skim milk prepared in PBST for 2 h at RT. Then, the membrane was incubated with specific primary antibodies overnight at 4°C. The next day, the membrane was washed three times with PBST (5 min per wash) to remove unbound primary antibodies. Afterwards, appropriate secondary antibodies labeled with HRP (abs20039, Absin, Shanghai, China) were added and incubated at RT for 1 h. Finally, signals were developed using a chemiluminescent substrate (KGC4602, KeyGen, China), and protein band images were captured by ChemiDoc Touch Imaging System (Bio-Rad Laboratories, Inc., Shanghai, China).

### Protease cleavage assay

For digestion, 1 µL aliquots of P29 mAb and P29 mAb^kbhb^ (both at 0.5 µg/µL) were each mixed with 20 µL of 0.25% trypsin (T1300, Solabao, China) and 29 µL of plasmin (0.05 µg/µL) (9001-90-5, Rhawn, China) and incubated at 37°C. Aliquots were removed after 1, 5, or 10 min of incubation and the reactions were immediately stopped by heating at 95°C for 5 min.

### Immunofluorescence assay

To detect the expression and localization of the endogenous P29 protein in *Echinococcus multilocularis* protoscolex (PSC) and alveolar hydatid cyst, respectively, the following procedures were performed. First, both PSCs and cysts were fixed in 4% polyformaldehyde at RT for 20 min, followed by three washes with PBS. PSCs were then embedded in 3% agarose at 45°C until solidification. The solidified agar blocks were placed on a freezing stage and sectioned into 4 μm slices using a cryostat microtome (CRYOSTAR NX50, Thermo). Afterwards, the sections were carefully transferred onto glass slides and air-dried. For cyst samples, after fixation, they were dehydrated, cleared, and embedded in paraffin using an embedding station (JB-P5, China). The resulting paraffin blocks were sectioned into 4 µm slices, which were transferred onto slides and dried at 60°C for 20 min. Next, all sections were processed identically. Paraffin sections were deparaffinized and rehydrated. Following three PBS washes to remove residual fixative, samples were permeabilized with 0.1% Tween-20 for 10 min at RT and washed again three times with PBS. To reduce nonspecific binding, sections were blocked with 3% BSA for 1 h at RT and washed three times with PBS (5 min per wash). After blocking, sections were incubated overnight at 4°C with appropriately diluted primary antibody. The next day, unbound primary antibodies were removed by three PBS washes. After gently removing excess liquid, sections were incubated with corresponding fluorescent secondary antibody for 1 h at RT in the dark, followed by three additional PBS washes (5 min per wash). Finally, slides were mounted with anti fluorescence quenching sealing agent containing DAPI (G1407, Servicebio, China). All images were acquired using an Upright Fluorescence Microscope (NIKON ECLIPSE C1, Japan).

### TUNEL staining

Apoptosis in paraffin-embedded alveolar hydatid cyst sections was analyzed by TUNEL staining using a TMR (red) TUNEL Cell Apoptosis Detection Kit (G1502, Servicebio, China), following the manufacturer’s protocol. Images were acquired using an upright fluorescence microscope (NIKON ECLIPSE C1, Japan).

### Histochemical staining

To evaluate therapeutic efficacy, twenty-seven C57BL/6 mice were intraperitoneally infected with PSCs. Three months post-infection, successful establishment of the AE mouse model was confirmed by abdominal ultrasound visualization of distinct alveolar hydatid cysts. The infected mice were then randomly divided into three treatment groups (n=9 per group) receiving weekly intravenous injections via the tail vein for eight weeks: Group 1 (Isotype IgG, control), Group 2 (P29 mAb), and Group 3 (P29 mAb^kbhb^), all at a dose of 5 mg/kg. At the experimental endpoint, all mice were euthanized, and alveolar hydatid cysts were collected from the abdominal cavity. Cysts were fixed in 4% paraformaldehyde at RT for 24 h, followed by paraffin embedding and sectioning (4 µm thickness) as described in the immunofluorescence assay methods. For histological analysis, serial sections were subjected to hematoxylin and eosin (H&E) staining and periodic acid-Schiff (PAS) staining. The H&E staining was performed as follows: sections were stained with hematoxylin for 5 min, rinsed in water, differentiated briefly in acid alcohol, rinsed in tap water to achieve bluing, and counterstained with eosin dye for 15 s. Following dehydration through a graded ethanol series and clearing in xylene, sections were mounted with neutral resin (10004160, SCRC, China). PAS staining was conducted strictly according to the manufacturer’s protocol of PAS staining kit (G1008, Servicebio, China). All stained tissue sections were photographed under an upright optical microscope (NIKON ECLIPSE E100, Japan).

### Surface plasmon resonance assay

The binding affinity between the P29-targeted antibodies and recombinant P29 protein was analyzed using OpenSPR Biomolecular Interaction Analyzer (Nicoya, Canada). Recombinant P29 protein was immobilized onto a high-sensitivity carboxyl sensor chip (SHG0626, Nicoya) via amine coupling. Briefly, the protein was diluted in PBS buffer to 20 µg/mL. The carboxyl groups on the chip surface were first activated with a freshly prepared mixture of 0.2 M carbodiimide (EDC) and 0.05M N-hydroxysuccinimide (NHS) (1:1 ratio) for 7 min at a flow rate of 10 μL/min. Subsequently, the protein solution was injected for 7 min to allow covalent coupling to the activated surface. Remaining active esters were then blocked by a 7-min injection of 1 M ethanolamine hydrochloride (pH 8.5). For kinetic analysis, both P29 mAb and P29 mAb^kbhb^ were serially diluted in PBS to concentrations ranging from 31.3 to 1000 nM. Antibody samples were injected over the protein-immobilized surface at a constant flow rate. Between sample injections, the sensor surface was regenerated using 50 mM glycine solution (pH 9.5). All interactions were monitored in real-time using the predefined “Kinetics/Affinity” assay template. The obtained data were processed and fitted to a 1:1 Langmuir binding model using TraceDrawer analysis software (Nicoya) to determine the association rate constant (ka), dissociation rate constant (kd), and equilibrium dissociation constant (KD).

### Mass spectrometry detection

The P29 mAb^kbhb^ protein sample (60 µg) was first separated using 10% SDS-PAGE. Following electrophoresis, the specific gel bands corresponding to the molecular weights of the antibody’s heavy chain and light chain were carefully excised. These excised gel pieces containing the target proteins were then subjected to subsequent mass spectrometry (MS) analysis, which was performed strictly in accordance with the detailed methodology provided in reference (Li et al., 2022).

### Statistics

In this study, randomization principles were rigorously applied to both animal grouping and microscopic field selection, with animals allocated using a random number table method and imaging fields selected through software-based randomization or systematic sampling strategies to ensure unbiased data collection. Throughout data acquisition and outcome assessment, we remained blinded to group assignments, with sample sizes determined based on preliminary experiments. All quantitative data were analyzed using GraphPad Prism (v10.0), presented as mean values ± standard error of mean (SEM), and between-group comparisons were performed using two-tailed Student’s t-tests with statistical significance defined as p < 0.05.

## Results

### Kbhb modification preserves the antigen-binding function of P29 mAb

**Figure 1:**
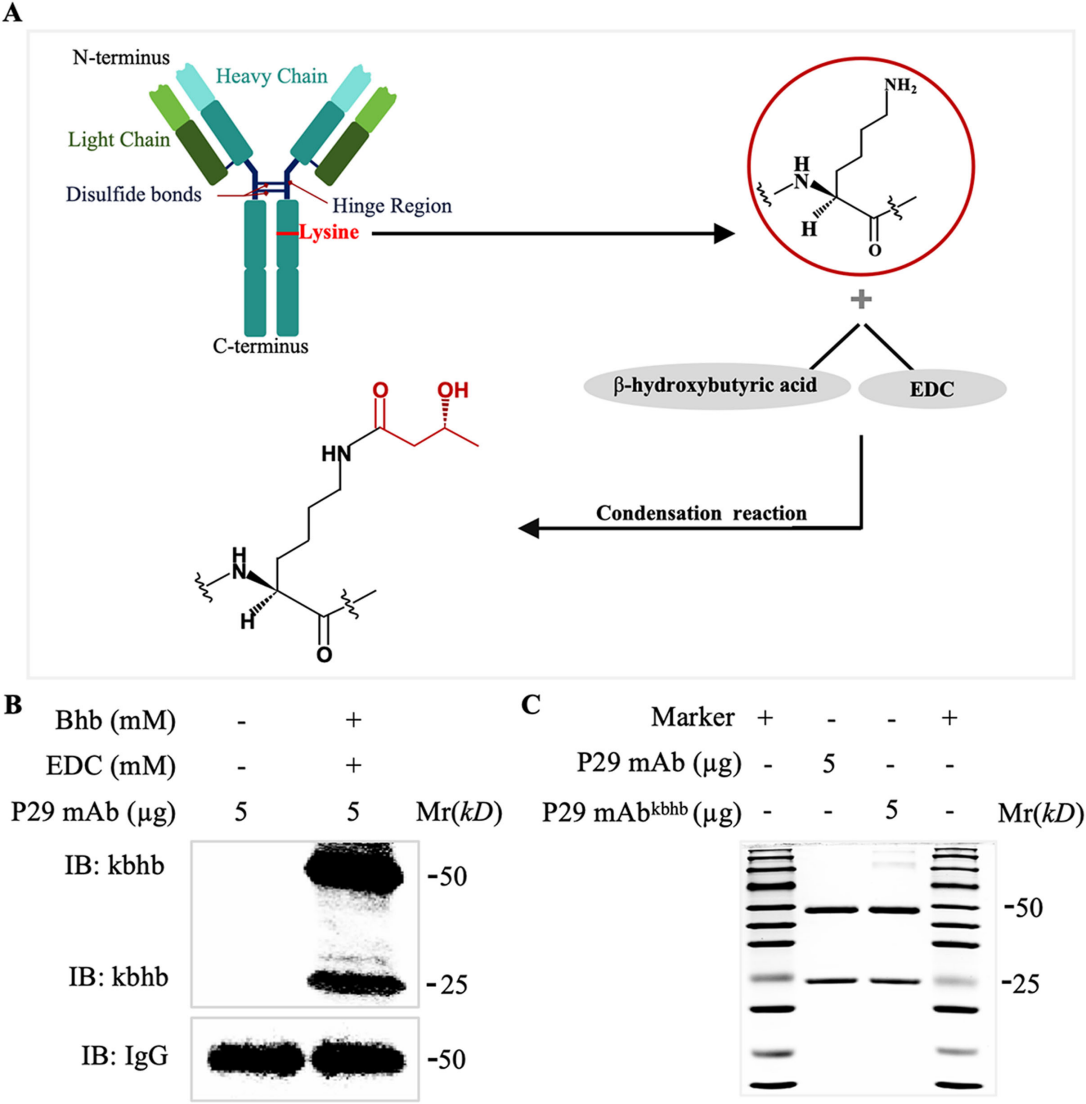
Preparation and verification of P29 mAb^kbhb^*in vitro*. **(A)** Schematic diagram illustrating the EDC-mediated random conjugation of the β-hydroxybutyryl **(kbhb)** group to multiple accessible lysine residues on the P29 mAb, resulting in a heterogeneous population of modified antibodies. The specific site depicted is for illustrative purposes only. **(B)** Western blot analysis confirming the kbhb modification using an anti-β-hydroxybutyryl-lysine antibody. **(C)** Coomassie brilliant blue staining verifying equal protein loading. EDC, 1-(3-dimethylaminopropyl) 3-ethylcarbodiimide hydrochloride.

**Figure 2:**
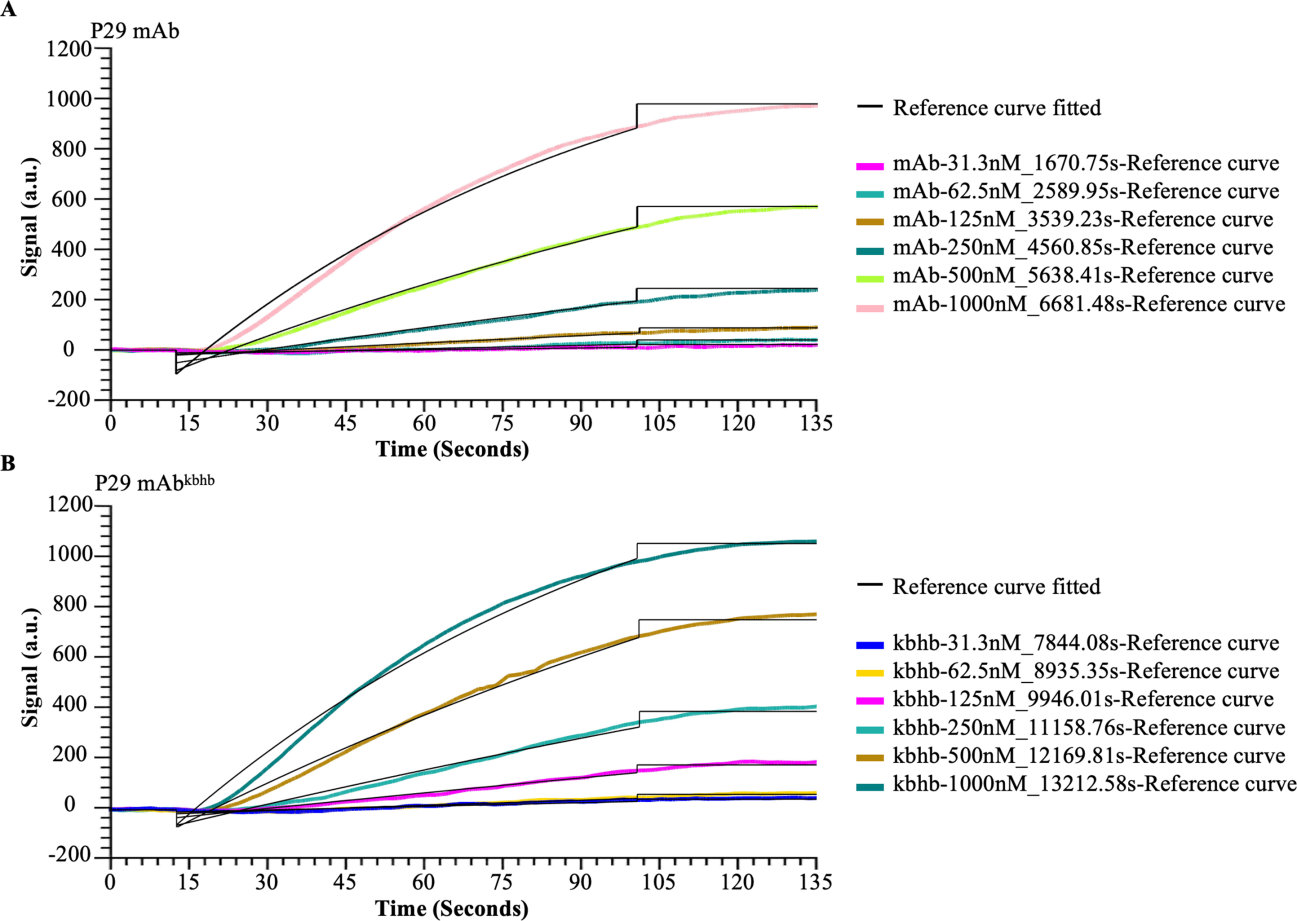
SPR binding kinetics of P29 mAb and P29 mAb^kbhb^ with recombinant P29 protein. **(A)** SPR binding kinetics of P29 mAb at different concentrations (31.3 nM, 62.5 nM, 125 nM, 250 nM, 500 nM, 1000 nM) with recombinant P29 protein. **(B)** SPR binding kinetics of P29 mAb^kbhb^ at different concentrations (31.3 nM, 62.5 nM, 125 nM, 250 nM, 500 nM, 1000 nM) with recombinant P29 protein.

**Figure 3:**
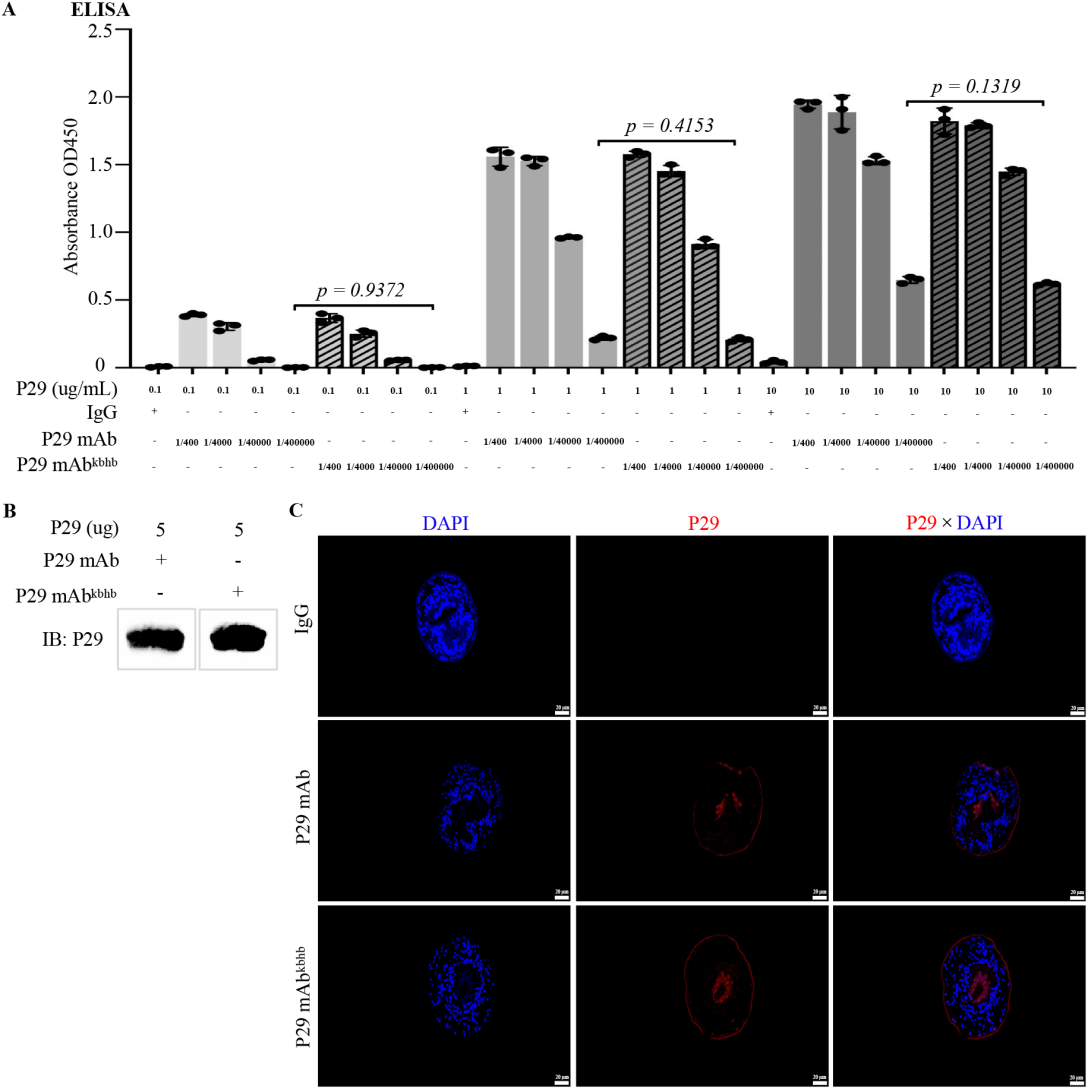
Antigen-binding characterization of P29 mAb^kbhb^*in vitro* and *in vivo*. **(A)***In vitro* antigen-binding analysis by ELISA. Serial dilutions (1:400 to 1:400,000) of P29 mAb^kbhb^ were tested against recombinant P29 protein at three coating concentrations (0.1, 1, and 10 µg/mL). IgG and wild-type P29 mAb served as negative and positive controls, respectively. Data are presented as mean ± SEM (n=3 independent experiments, each performed in triplicate). Statistical comparisons between P29 mAb^kbhb^ and wild-type P29 mAb at each antigen concentration were analyzed by two-tailed Student’s t-test, with exact p-values indicated directly on the figure. **(B)** Western blot analysis. Antigen recognition capability of P29 mAb^kbhb^ was assessed by immunoblotting using recombinant P29 protein. **(C)***In vivo* antigen detection in *Echinococcus multilocularis* PSC. Immunofluorescence staining of *Echinococcus multilocularis* PSC tissue sections showing P29 antigen (red) recognition by P29 mAb^kbhb^. Scale bar, 20 µm.

To investigate the functional consequences of kbhb modification on antibody activity, we first generated P29 mAb^kbhb^ via 1-(3-(dimethylamino) propyl)-3-ethylcarbodiimide hydrochloride (EDC)-mediated chemical conjugation (**Figure 1A**). Successful modification was confirmed by mass spectrometry, which showed the expected mass shifts, and by immunoblotting with an anti-β-hydroxybutyryl-lysine antibody (**Figure 1B**, **Supplementary d1**). Equivalent protein concentrations of modified and unmodified antibodies were verified by BCA assay and Coomassie staining (**Figure 1C**). We then systematically evaluated antigen-binding capability of P29 mAb^kbhb^ through multiple complementary approaches. Surface plasmon resonance (SPR) analysis revealed that P29 mAb^kbhb^ maintained picomolar affinity for recombinant P29 protein (KD = 343 pM) comparable to unmodified P29 mAb (KD = 265 pM), with no statistically significant difference in binding kinetics (**Figure 2**; **Table 1**). This preservation of high-affinity interaction was further supported by immunoblotting and enzyme-linked immunosorbent assay (ELISA), both demonstrating equivalent target recognition (**Figures 3A, B**). Importantly, when assessing biological relevance through immunofluorescence staining of *Echinococcus multilocularis* PSC tissues and alveolar hydatid cysts, P29 mAb^kbhb^ exhibited identical antigen labeling patterns to its unmodified counterpart, particularly in critical PSC structures like the tegument, rostellum, and suckers (**Figure 3C**; **Supplementary Figure S1**). Together, these consistent findings across biochemical, biophysical, and biological assays demonstrate that kbhb modification preserves the structural integrity of antigen-binding sites and the functional recognition of both recombinant and native P29 antigen, while conferring potential stability advantages previously reported.

**Table 1:** Comparison of dissociation constants between P29 mAb and P29 mAb^kbhb^ binding to P29 protein. .

Samples	ka (1/M*s)	kd (1/s)	KD (M)
P29 mAb	1.16×10^-4^	3.08×10^-6^	2.65×10^-10^
P29 mAb^kbhb^	1.03×10^-4^	3.54×10^-6^	3.43×10^-10^

KD, dissociation constant; ka, association rate constant; kd, dissociation rate constant.

### Enhanced proteolytic stability and improved pharmacokinetics of β-hydroxybutyrylated P29 mAb

**Figure 4:**
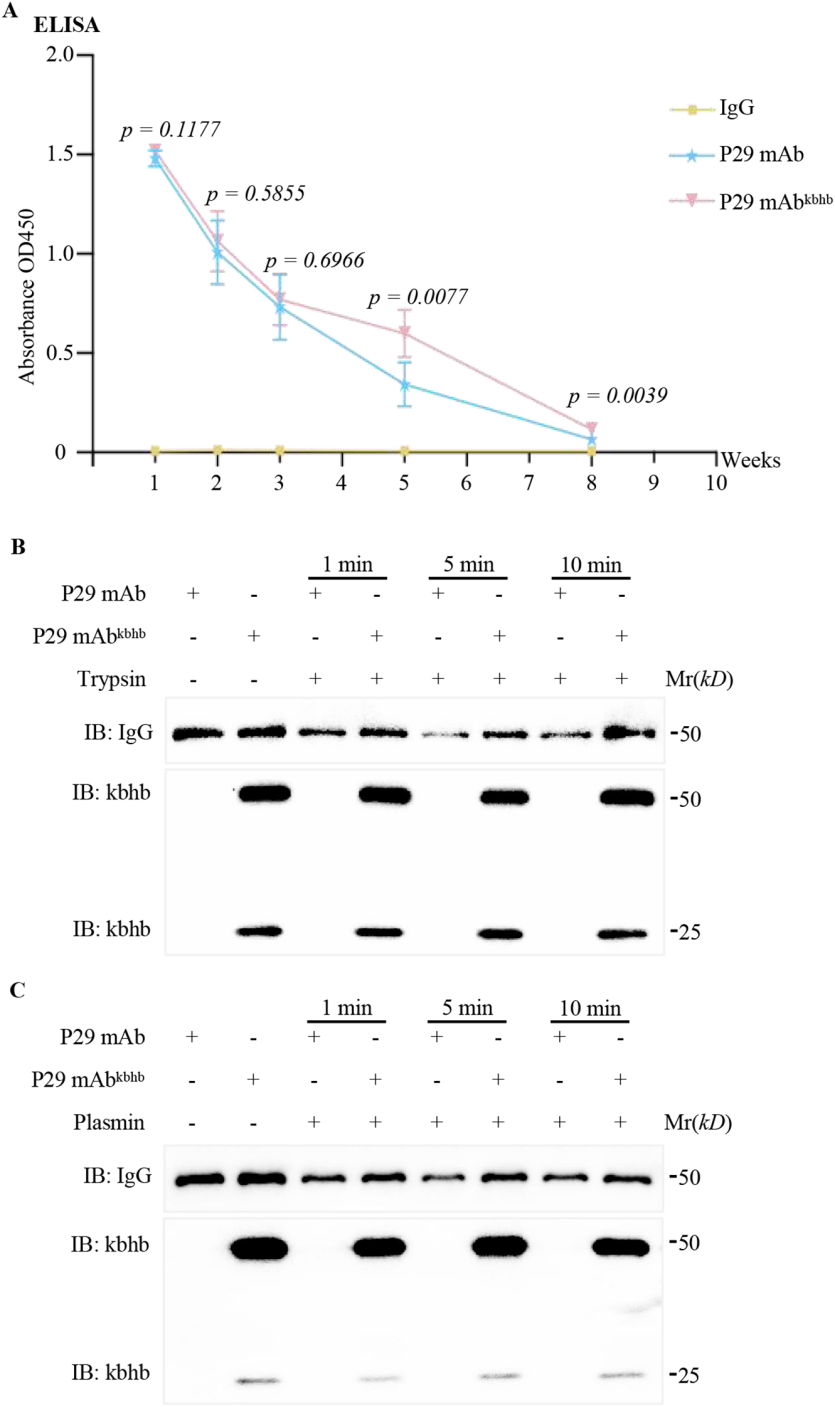
Stability evaluation of P29 mAb and P29 mAb^kbhb^*in vivo* and *in vitro*. **(A)***In vivo* pharmacokinetic analysis of P29 mAb and P29 mAb^kbhb^. Serum antibody concentrations were quantified by ELISA at the indicated time points (1–8 weeks) following intravenous administration (1 µg/mL P29-coated plates, n=5 mice per group). Data are presented as mean ± SEM. Statistical significance was determined by two-tailed Student’s t-test, with exact p-values indicated directly on the figure. **(B, C)** Proteolytic resistance assay. Trypsin (Plasmin) susceptibility of P29 mAb and P29 mAb^kbhb^ was assessed by immunoblotting after incubation at 37°C for 1, 5, and 10 minutes.

Having established that kbhb modification preserves the antigen-binding capacity of P29 mAb, we proceeded to evaluate its impact on antibody stability through a series of *in vivo* and *in vitro* assays. Pharmacokinetic analysis revealed a time-dependent enhancement in the circulatory persistence of the modified antibody. Following intravenous administration in C57BL/6 mice (n=5/group), longitudinal monitoring of serum concentrations by ELISA demonstrated that P29 mAb^kbhb^ maintained slightly elevated levels over the unmodified antibody during the initial 1–4 weeks, though these differences did not reach statistical significance. A pronounced and statistically significant divergence emerged at the 5-week timepoint, where the serum concentration of P29 mAb^kbhb^ was approximately 1.75-fold higher than that of the unmodified antibody. This significant difference in persistence was maintained through the terminal measurement at 8 weeks (**Figure 4A**).

To explore the potential mechanism underlying this pharmacokinetic improvement, we subjected the antibodies to *in vitro* proteolytic challenge. Immunoblot analysis after controlled trypsin or plasmin digestion demonstrated that P29 mAb^kbhb^ exhibits markedly increased resistance to enzymatic degradation compared to the unmodified control (**Figures 4B, C**). This enhanced proteolytic stability provides a plausible explanation for the extended serum half-life observed *in vivo*.

Taken together, these findings demonstrate that kbhb modification confers three critical pharmacological advantages: (1) preservation of antigen recognition capacity as previously established, (2) moderately prolonged *in vivo* circulation time, and (3) enhanced resistance to proteolytic degradation. The increased metabolic stability is particularly noteworthy given the harsh proteolytic environment encountered by therapeutic antibodies in circulation. While the precise structural mechanisms underlying this protease resistance require further investigation through crystallographic or hydrogen-deuterium exchange studies, our data suggest that kbhb-induced conformational stabilization or shielding of vulnerable cleavage sites may be contributing factors.

From a translational perspective, these properties position P29 mAb^kbhb^ as an improved candidate for AE treatment, where extended antibody persistence could enhance therapeutic efficacy by sustaining serum levels. The successful preservation of antigen recognition combined with improved metabolic stability represents a promising step in antibody engineering for parasitic diseases, indicating that kbhb modification may offer a generally applicable approach to optimizing the pharmacokinetic profile of therapeutic antibodies.

### Therapeutic potential of P29 mAb^kbhb^ in AE treatment

**Figure 5:**
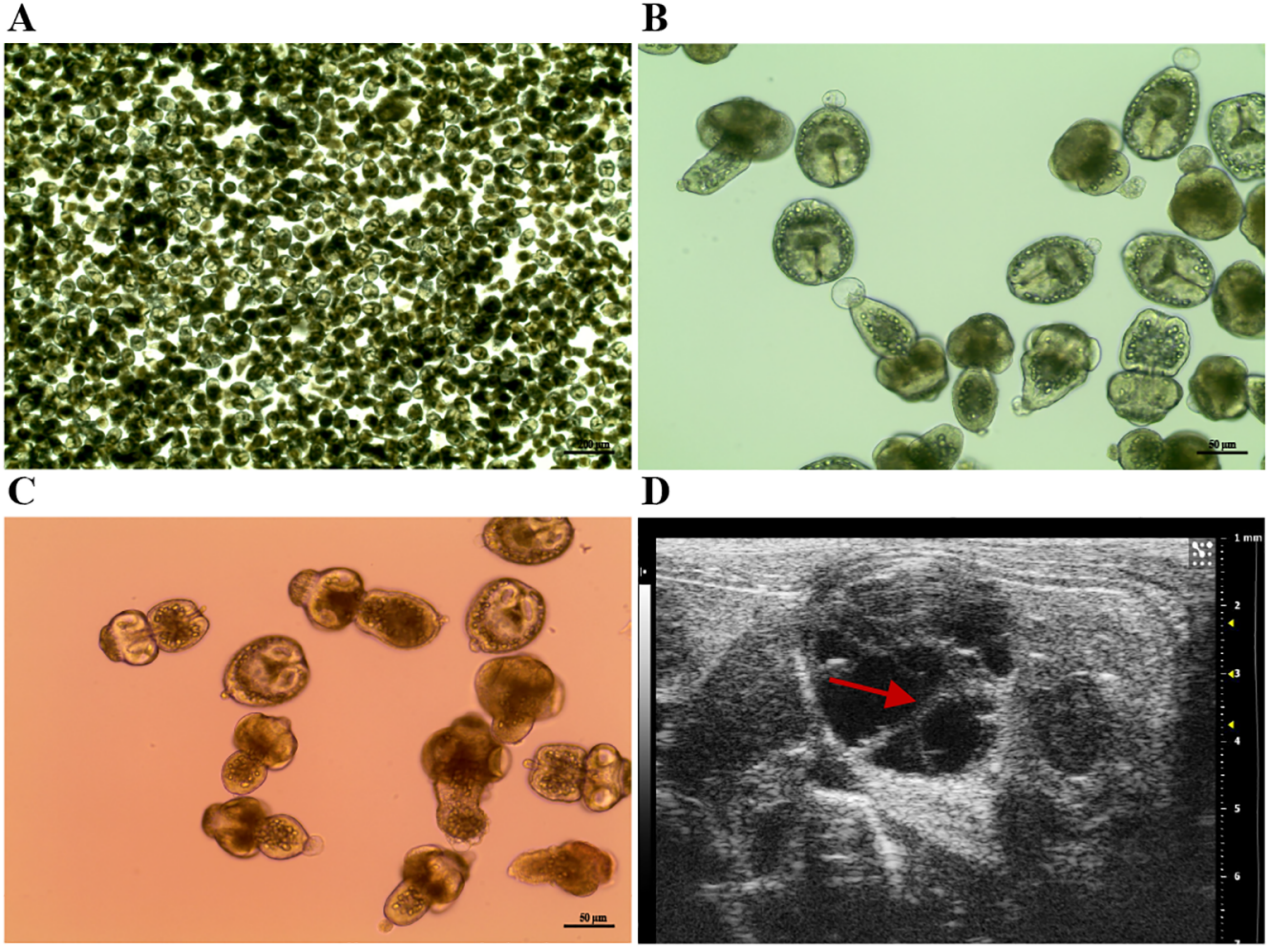
Establishment and characterization of a murine AE model. **(A)** PSC morphology. Low-magnification micrograph of *Echinococcus multilocularis* PSC. Scale bar, 200 µm. **(B)** High-resolution PSC imaging. Magnified view of PSCs showing detailed morphological features. Scale bar, 50 µm. **(C)** Viability assessment. PSC viability confirmed by eosin exclusion staining (viable PSCs exclude eosin dye). **(D)***In vivo* disease progression. A representative abdominal ultrasound image acquired at 3 months post-inoculation, revealing characteristic cystic-solid hypoechoic lesions (indicated by red arrow).

**Figure 6:**
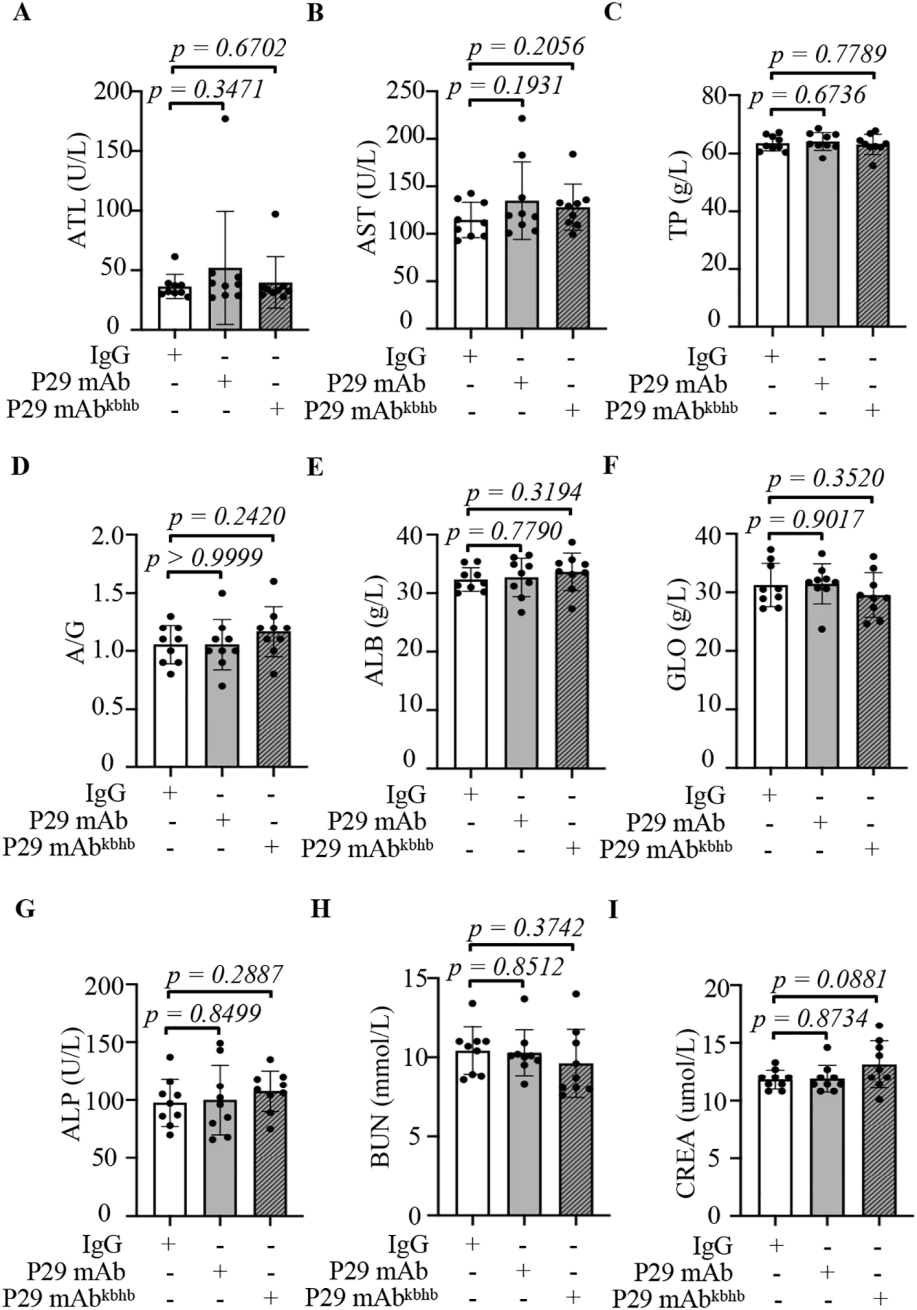
Safety evaluation of P29 mAb^kbhb^ on hepatic and renal functions. **(A-I)** Serum biochemical analysis. Hepatic and renal function parameters in PSC-infected mice following 8-week treatment with IgG (n=9), P29 mAb (n=9), and P29 mAb^kbhb^ (n=9). ATL, alanine aminotransferase; AST, Aspartate aminotransferase; TP, Total protein; ALB, Albumin; GLO, Globulin; A/G, the Albumin and Globulin Ratio; ALP, Alkaline Phosphatase; BUN, blood urea nitrogen; CREA, creatinine. Data are presented as mean ± SEM. Statistical significance was determined by two-tailed Student’s t-test, with exact p-values indicated directly on the figure.

**Figure 7:**
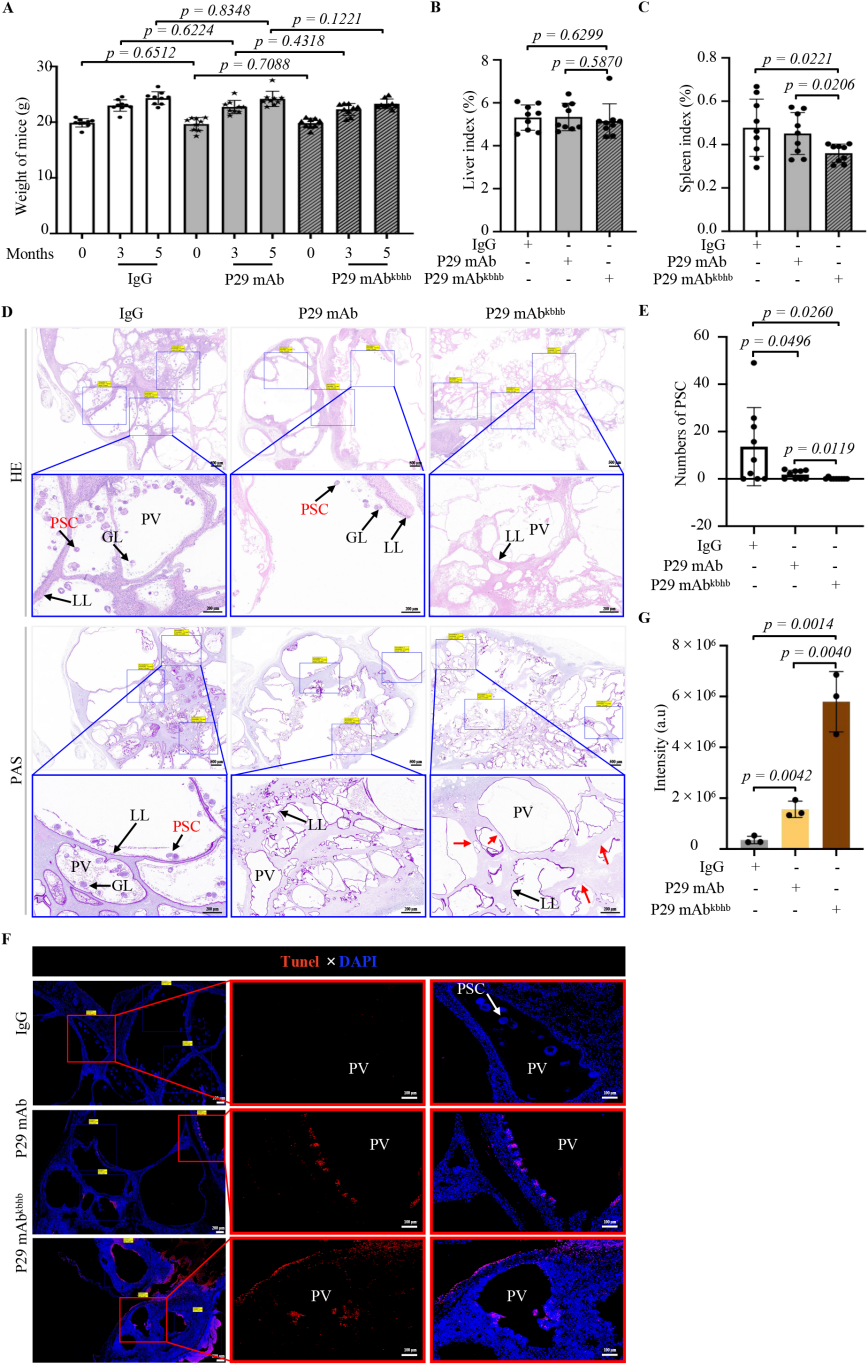
Therapeutic efficacy of P29 mAb^kbhb^ in a murine model of AE. **(A)** Longitudinal monitoring of body weight in PSC-infected mice. Body weights were recorded at the indicated timepoints (0, 3, and 5 months post-infection) in mice treated with IgG (n=9), P29 mAb (n=9), or P29 mAb^kbhb^ (n=9). Treatment was initiated at 3 months post-infection. **(B, C)** Organ indices at study endpoint. Liver index **(B)** and spleen index **(C)** were measured in PSC-infected mice at 5 months post-infection following treatment with IgG (n=9), P29 mAb (n=9), or P29 mAb^kbhb^ (n=9). **(D-G)** Parasitic burden assessment. **(D)** Histopathological analysis. Upper panel: HE staining showing structural features of alveolar hydatid cyst. Lower panel: PAS staining highlighting carbohydrate-rich layers. Arrows indicate: - Black: Key structures (GL = germinal layer, LL = laminated layer, PV = parasitic vesicle). - Red: Degenerative changes in the capsule wall. **(E)** PSC regenerative capacity. PSC numbers were quantified in alveolar hydatid cyst sections from AE model mice treated with IgG (n=9), P29 mAb (n=9), or P29 mAb^kbhb^ (n=9). Three microscopic fields were analyzed per sample. **(F)** Representative histological images of TUNEL staining in alveolar hydatid cyst tissues. Cyst sections from AE model mice treated with IgG, P29 mAb, or P29 mAb^kbhb^ are shown. For each treatment group, low-magnification views (left panels; scale bars = 200 μm) display overall tissue architecture and TUNEL + cell distribution, with boxed regions indicating the areas selected for high-magnification imaging (right panels; scale bars = 100 μm). The high-magnification images highlight detailed morphological features of apoptotic cells (magenta staining). **(G)** Quantitative analysis of TUNEL staining fluorescence intensity. Apoptotic levels were assessed in alveolar hydatid cyst sections from AE model mice treated with IgG (n=3), P29 mAb (n=3), or P29 mAb^kbhb^ (n=3). Three mice were randomly selected from each group (n=9) for analysis, with three microscopic fields quantified per sample. All data are presented as mean ± SEM. Statistical significance was determined by two-tailed Student’s t-test, with exact p-values indicated directly on the figure. PSC, Protoscolex. HE, hematoxylin and eosin; PAS, Periodic Acid-Schiff; GL, germinal layer; LL, laminated layer; PV, parasitic vesicle.

Our investigation into the therapeutic effects of P29 mAb^kbhb^ began with establishing the murine AE model through intraperitoneal inoculation of viable *Echinococcus multilocularis* PSCs. Successful infection was confirmed after three months by abdominal ultrasound imaging, which revealed characteristic alveolar hydatid cysts in the peritoneal cavity (**Figure 5**). We then evaluated the treatment efficacy by administering weekly doses (5 mg/kg) of either isotype IgG (control), native P29 mAb, or P29 mAb^kbhb^ to infected mice over an eight-week period. Comprehensive assessment of treatment outcomes demonstrated several key findings regarding P29 mAb^kbhb^’s performance. Importantly, all treatment groups showed comparable profiles in hepatic and renal function markers, body weight measurements, and liver indices, indicating the modified antibody maintains a favorable safety profile similar to its unmodified counterpart (**Figure 6** and **Figures 7A, B**). However, P29 mAb^kbhb^ treatment resulted in significantly reduced splenomegaly compared to control groups, suggesting an enhanced ability to modulate infection-associated inflammatory responses (**Figure 7C**). While the overall weight of alveolar hydatid cysts showed only a non-significant decreasing trend in the P29 mAb^kbhb^ group, detailed histopathological examination revealed more profound therapeutic effects. The modified antibody treatment resulted in severely impaired PSC regeneration within the alveolar hydatid cysts, accompanied by increased apoptosis of cyst wall cells and expanded necrotic areas (**Figures 7D-G**; **Supplementary Figure S2**). These morphological alterations were associated with a significant reduction in PSC regenerative capacity, demonstrating the enhanced efficacy of P29 mAb^kbhb^ in impairing both the cellular activity and structural integrity of alveolar hydatid cysts. The improved anti-parasitic activity of P29 mAb^kbhb^ likely stems from its modified biochemical properties. Our pharmacokinetic studies showed that the kbhb modification prolongs the antibody’s serum persistence. This extended circulation time presumably allows for greater target engagement and more sustained therapeutic action against the parasite. The current findings complement those observations by showing how these pharmacokinetic advantages translate to enhanced biological efficacy against established echinococcal infections. These results collectively position P29 mAb^kbhb^ as a promising therapeutic candidate for AE, combining the safety profile of the native antibody with improved anti-parasitic activity. The kbhb modification appears to potentiate treatment efficacy through multiple mechanisms, including prolonged target exposure and direct enhancement of anti-metacestode effects, without introducing detectable toxicity. This approach of targeted antibody modification may offer new opportunities for improving therapeutics against chronic parasitic infections where treatment durability is often limiting.

## Discussion

This study systematically investigated the functional impact of kbhb modification on a monoclonal antibody targeting the P29 protein of *Echinococcus multilocularis*. The results demonstrate that while preserving the intrinsic antigen affinity of the antibody (**Figures 2**, **3**; **Supplementary Figure S3**; **Supplementary d1**), this modification markedly enhanced its resistance to proteolytic degradation (**Figures 4B, C**) and, to a certain extent, improved its pharmacokinetic properties *in vivo*, ultimately exhibiting superior therapeutic potential compared to the unmodified antibody.

Delving into the mechanism, the enhancement of antibody stability by kbhb modification is based on a sophisticated alteration of the physicochemical properties of lysine residues. Our prior research on a model peptide (S-2251 oligopeptide) provided crucial clues for this (Li et al., 2022): kbhb modification of a key lysine in this peptide completely blocked its cleavage by trypsin/plasmin. This finding aligns well with the fundamental principle of protease substrate recognition-the catalytic activity of serine proteases like trypsin/plasmin highly depends on the specific recognition of positively charged lysine/arginine residues in substrates (Friberger et al., 1978; More et al., 2021). We speculate that kbhb modification disrupts this process synergistically: the modification neutralizes the positive charge of lysine, weakening its electrostatic attraction to the protease active site; concurrently, the introduced kbhb group creates a steric hindrance, physically impeding the effective formation of the enzyme-substrate complex.

It is worth exploring in greater depth how this stabilization mechanism, grounded in molecular interface engineering, contributes to the therapeutic advantage. It should be noted that the inherent heterogeneity of the current kbhb modification strategy may lead to a limited proportion of highly stable subpopulations within the antibody pool. This could be a potential reason why the overall pharmacokinetic benefit of this group was not maximized, thereby restricting further optimization of its therapeutic efficacy to the highest level. Specifically, regarding efficacy endpoints, compared to the negative and positive control groups, the kbhb-modified antibody group showed a downward trend in cyst weight, although it did not reach statistical significance. More importantly, this group demonstrated statistically significant improvements in two key aspects: inhibiting the regenerative capacity of protoscoleces—a critical component within the cysts—and effectively inducing apoptosis of the cyst wall cells. Although experimental design limitations prevented precise correlation analysis at the individual level, this group-level covariation suggests that the moderate extension of serum persistence may have supported the attainment of the aforementioned therapeutic effects. In other words, the molecular stability conferred by kbhb modification is likely a beneficial factor linking the improved pharmacokinetics to the enhanced treatment outcome. Furthermore, developing site-specific modification techniques to overcome heterogeneity holds promise for fully unleashing its therapeutic potential in the future.

Placing our study in a broader biological context, the kbhb modification investigated here represents just one component of the extensive lysine modification network. This network also encompasses a series of chemically distinct modifications derived from key metabolites, including acetylation, malonylation, lactylation, propionylation, butyrylation, and succinylation (Chen et al., 2007; Peng et al., 2011; Ding et al., 2022; Shen et al., 2022; Bai et al., 2024; Zhou et al., 2025). Since the initial reports of kbhb and crotonylation in 2011 (Tan et al., 2011; Xie et al., 2016), proteomic studies have continued to reveal the remarkable diversity of lysine modifications. For example, Huang et al (Huang et al., 2021). identified over 3,000 kbhb modification sites in HEK 293 cells in a single study; subsequent research has confirmed that such modifications are widely present across the proteomes of diverse organisms (Contreras-de la Rosa et al., 2022; Zheng et al., 2023; Song et al., 2025). These findings collectively outline a complex “landscape of lysine modifications,” in which different modifications exert precise and multifaceted regulatory control over protein function by modulating their charge states, spatial conformations, and intermolecular interactions (Bai et al., 2024; Zhou et al., 2025). With this context, our work translates fundamental biological discoveries into a practical antibody engineering strategy, demonstrating the feasibility of using a lysine modification to modulate the function of therapeutic proteins.

From a technological development perspective, the antibody kbhb modification strategy developed in this study offers a valuable supplementary approach for antibody drug stabilization engineering. Distinct from conventional strategies that increase molecular size or modulate FcRn interactions (Pan et al., 2018; Li et al., 2019; Wuhrer et al., 2021; Gao et al., 2023; García-Alija et al., 2023), kbhb modification attempts to directly intervene in the protease-antibody recognition process by targeting the physicochemical nature of molecular interface interactions. Unlike PEGylation, which carries potential immunogenicity risks or may interfere with target binding, kbhb is derived from endogenous metabolites, suggesting superior biocompatibility potential. This study confirms that kbhb modification markedly enhances antibody stability while fully retaining its antigen-binding capacity, offering a new perspective for addressing the common challenge in traditional modification methods where “stability enhancement” and “functional retention” are often difficult to balance. Compared to Fc region modifications requiring complex genetic manipulation, kbhb modification, based on an efficient chemical conjugation route, offers advantages of operational simplicity and ease of scaling for industrial application.

While acknowledging the potential of the kbhb modification strategy, its technical optimization directions and potential risks must be carefully considered. Although the current random conjugation-based kbhb modification still requires optimization regarding product homogeneity, its demonstrated unique mechanistic advantages and application potential indicate that it may represent a worthwhile direction to explore in the evolution of antibody stabilization strategies from “macroscopic property modulation” towards “fine molecular interface design”. Simultaneously, any covalent modification of proteins carries potential risks, and lysine kbhb modification is no exception. The modification could alter the native conformation of the antibody, potentially inducing aggregation and thereby increasing its immunogenicity. Although no significant immune response was detected during the observation period of this study, long-term assessment of this risk is indispensable.

Based on the above analysis, we propose the following future research directions: The primary task is to develop site-specific kbhb modification techniques to overcome the heterogeneity issues caused by current random modification. Secondly, conducting parallel comparative studies with other lysine modifications, such as acetylation, will help elucidate the unique functions and mechanisms of kbhb modification. Particularly important is establishing experimental models capable of synchronously assessing individual-level pharmacokinetics and pharmacodynamics, to provide direct evidence for understanding the exposure-response relationship between pharmacokinetic improvement and therapeutic effect. Finally, comprehensive validation in large animal models will be an essential step for evaluating its clinical prospects.

## Conclusion

This study confirms that kbhb modification contributes to the optimization of the stability and *in vivo* performance of the anti-E. multilocularis P29 antibody, providing a promising new strategy for the development of anti-echinococcosis antibody drugs. The research not only revealed the potential mechanism of action of this modification and preliminarily clarified its associative pathway from molecular stability to therapeutic effect, but also objectively analyzed its technical advantages and development directions, laying a solid foundation for subsequent research.

## Data Availability

The complete dataset supporting this research, including all numerical values underlying **Figures 1**–**7** and **Table 1**, has been provided in the main paper, other key data were compiled in **Figure S1–S3**. Mass spectrometry data are provided in **Supplementary d1**. All primary experimental data and materials are maintained by the corresponding authors and will be made available upon reasonable request.

## Supplementary material

Supplementary Figure S1 Antigen-binding characterization of P29 mAbkbhb in alveolar hydatid cyst. Alveolar hydatid cyst tissue sections were stained with P29 mAbkbhb (red) to detect P29 antigen distribution. Nuclei were counterstained with DAPI (blue). For each treatment group, low-magnification views (left panels; scale bars = 500 μm) display the overall tissue architecture and antigen distribution patterns, with boxed regions indicating areas selected for high-magnification imaging (right panels; scale bars = 50 μm).

Supplementary Figure S2 Gross morphology and wet weight of alveolar hydatid cysts. **(A)** Representative images of alveolar hydatid cysts isolated from AE model mice following 8-week treatment with IgG (n=9), P29 mAb (n=9), or P29 mAbkbhb (n=9). **(B)** Quantitative analysis of cyst wet weight. Data are presented as mean ± SEM. Statistical significance was determined by two-tailed Student's t-test, with exact p-values indicated directly on the figure.

Supplementary Figure S3 Computational prediction of P29 mAb complementarity-determining regions (CDRs). **(A)** Structural model of P29 mAb variable domains. Predicted using SAbPred server with North/AHo numbering scheme, Light pink represent Variable light chain (VL), Steel blue represent Variable heavy chain (VH), Midnight blue represent Complementarity-determining regions (CDRs).
