# Supplementary material for: Enhancing the structural stability of P29-targeted monoclonal antibodies via β-hydroxybutyrylation modification improves their therapeutic performance in alveolar echinococcosis
Source: Front Cell Infect Microbiol. 2026 Jan 5;15:1716047. doi: 10.3389/fcimb.2025.1716047 (PMC12812953; doi:10.3389/fcimb.2025.1716047)

Sequence: TSTSPIVKSFNR, K8-Kbhb (86.03680 Da)
Charge: +2, Monoisotopic m/z: 711.88226 Da (-0.75 mmu/-1.06 ppm), MH+: 1422.75724 Da, RT: 49.0156 min,
Identified with: Sequest HT (v1.17); XCorr:2.37,
Fragment match tolerance used for search: 0.02 Da
Fragments used for search: $-H_2O$; y; $-NH_3$; y; b; b; $-H_2O$; b; $-NH_3$; y

Fragment Matches

Value Type: Theo. Mass [Da]

Ion Series | Neutral Losses | Precursor Ions | Internal Fragments

#1	b^+	b^{2+}	Seq.	y^+	y^{2+}	#2
1	102.05496	51.53112	T			12
2	189.08698	95.04713	S	1321.71106	661.35917	11
3	290.13466	145.57097	T	1234.67903	617.84315	10
4	377.16669	189.08698	S	1133.63135	567.31932	9
5	474.21945	237.61337	P	1046.59933	523.80330	8
6	587.30352	294.15540	I	949.54656	475.27692	7
7	686.37193	343.68960	V	836.46250	418.73489	6
8	900.50369	450.75549	K-Kbhb	737.39408	369.20068	5
9	987.53572	494.27150	S	523.26232	262.13480	4
10	1134.60414	567.80571	F	436.23029	218.61879	3
11	1248.64706	624.82717	N	289.16188	145.08458	2
12			R	175.11895	88.06311	1

VL+CL:
DIVMTQSPLSLPVSLGDQASISCRSSQSIVHSNGNTYLEWYLQKPGQSPKLLIYKVSNRFSGVPDRFSGSGSGTDFTLKISRVEAEDLGVYY
CFQGSHVPYTFGGGTKLEIKRADAAPTVSIFPPSSEQLTSGGASVVCFLNNFYPKDINVKWKIDGSERQNGVLNSWTDQDSKDSTYSMSS
TLTLTKDEYERHNSYTCEATHKTSTSPIVKSFNRNEC

VL+CL: Variable region of light chain + Constant region of light chain

Fragment Spectrum

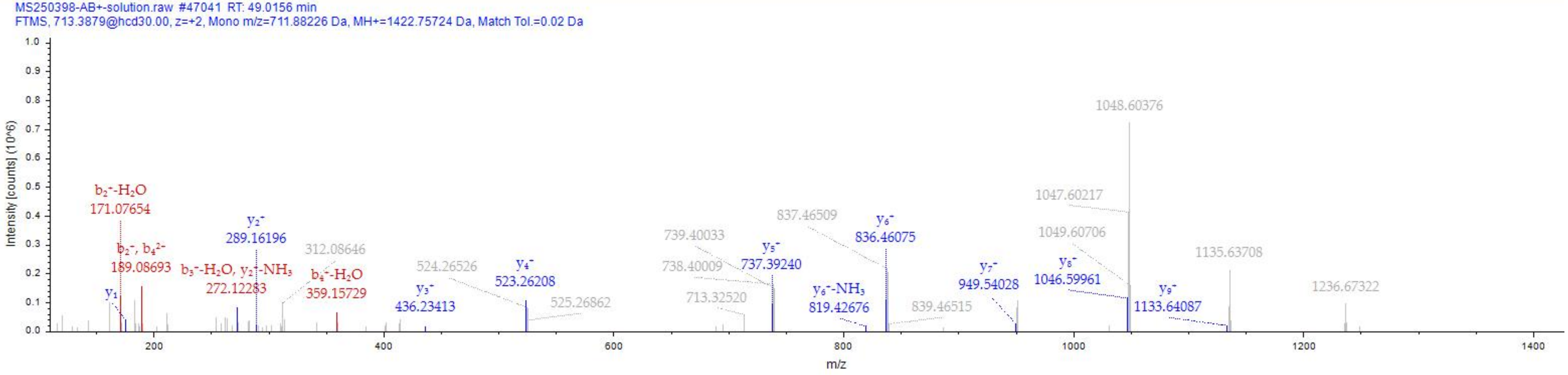

Sequence: LLIYKVSNR, K5-Kbhb (86.03680 Da)
Charge: +2, Monoisotopic m/z: 596.35786 Da (-0.58 mmu/-0.97 ppm), MH+: 1191.70845 Da, RT: 54.5431 min,
Identified with: Sequest HT (v1.17); XCorr:2.51,
Fragment match tolerance used for search: 0.02 Da
Fragments used for search: -H_2O; y; -NH_3; y; b; b; -H_2O; b; -NH_3; y

Fragment Matches

Value Type: Theo. Mass [Da]

Ion Series | Neutral Losses | Precursor Ions | Internal Fragments

#1	b^+	b^{2+}	Seq.	y^+	y^{2+}	#2
1	114.09134	57.54931	L			9
2	227.17540	114.09134	L	1078.62554	539.81641	8
3	340.25947	170.63337	I	965.54148	483.27438	7
4	503.32280	252.16504	Y	852.45741	426.73234	6
5	717.45456	359.23092	K-Kbhb	689.39408	345.20068	5
6	816.52297	408.76513	V	475.26232	238.13480	4
7	903.55500	452.28114	S	376.19391	188.60059	3
8	1017.59793	509.30260	N	289.16188	145.08458	2
9			R	175.11895	88.06311	1

VL+CL:
DIVMTQSPLSLPVSLGDQASISCRSSQSIVHSNGNTYLEWYLQKPGQSPKLLIYKVSNRFSGVPDRFSGSGSGTDFTLKISRVEAEDLGVY
YCFQGSHVPYTFGGGTKLEIKRADAAPTVSIFPPSSEQLTSGGASVVCFLNNFYPKDINVKWKIDGSERQNGVLNSWTDQDSKDSTYSM
SSTLTLTKDEYERHNSYTCEATHKTSTSPIVKSFNRNEC

VL+CL: Variable region of light chain + Constant region of light chain

Fragment Spectrum

MS250398-AB+-solution.raw #55046 RT: 54.5431 min
FTMS, 596.3575@hcd30.00, z=+2, Mono m/z=596.35786 Da, MH+=1191.70845 Da, Match Tol.=0.02 Da

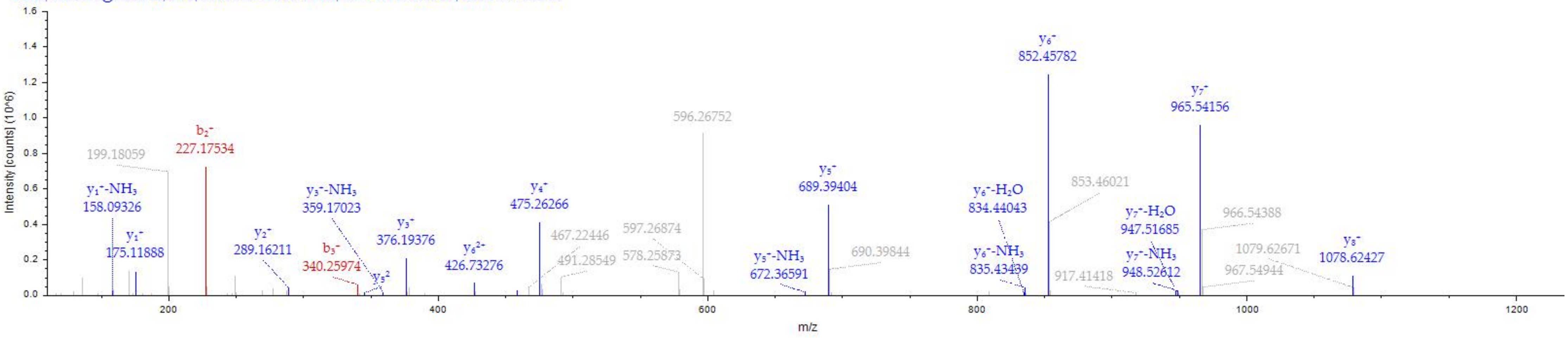

Sequence: WKIDGSER, K2-Kbhb (86.03680 Da)
Charge: +2, Monoisotopic m/z: 538.77174 Da (-0.46 mmu/-0.85 ppm), MH+: 1076.53620 Da, RT: 42.5684 min,
Identified with: Sequest HT (v1.17); XCorr:1.94,
Fragment match tolerance used for search: 0.02 Da
Fragments used for search: $-H_2O$; y; $-NH_3$; y; b; b; $-H_2O$; y

Fragment Matches

Value Type: Theo. Mass [Da]

Ion Series | Neutral Losses | Precursor Ions | Internal Fragments

#1	b^+	b^{2+}	Seq.	y^+	y^{2+}	#2
1	187.08659	94.04693	W			8
2	401.21835	201.11281	K-Kbhb	890.45781	445.73254	7
3	514.30242	257.65485	I	676.32604	338.66666	6
4	629.32936	315.16832	D	563.24198	282.12463	5
5	686.35082	343.67905	G	448.21504	224.61116	4
6	773.38285	387.19506	S	391.19357	196.10043	3
7	902.42544	451.71636	E	304.16155	152.58441	2
8			R	175.11895	88.06311	1

VL+CL:
DIVMTQSPLSLPVSLGDQASISCRSSQSIVHSNGNTYLEWYLQKPGQSPKLLIYKVSNRFSGVPDRFSGSGSGTDFTLKISRVEAEDLGVY
YCFQGSHVPYTFGGGTKLEIKRADAAPTVSIFPPSSEQLTSGGASVVCFLNNFYPKDINVKWKIDGSERQNGVLNSWTDQDSKDSTYSM
SSTLTLTKDEYERHNSYTCEATHKTSTSPIVKSFNRNEC

VL+CL: Variable region of light chain + Constant region of light chain

Fragment Spectrum

MS250398-AB+-solution.raw #39524 RT: 42.5684 min
FTMS, 540.2977@hcd30.00, z=+2, Mono m/z=538.77174 Da, MH+=1076.53620 Da, Match Tol.=0.02 Da

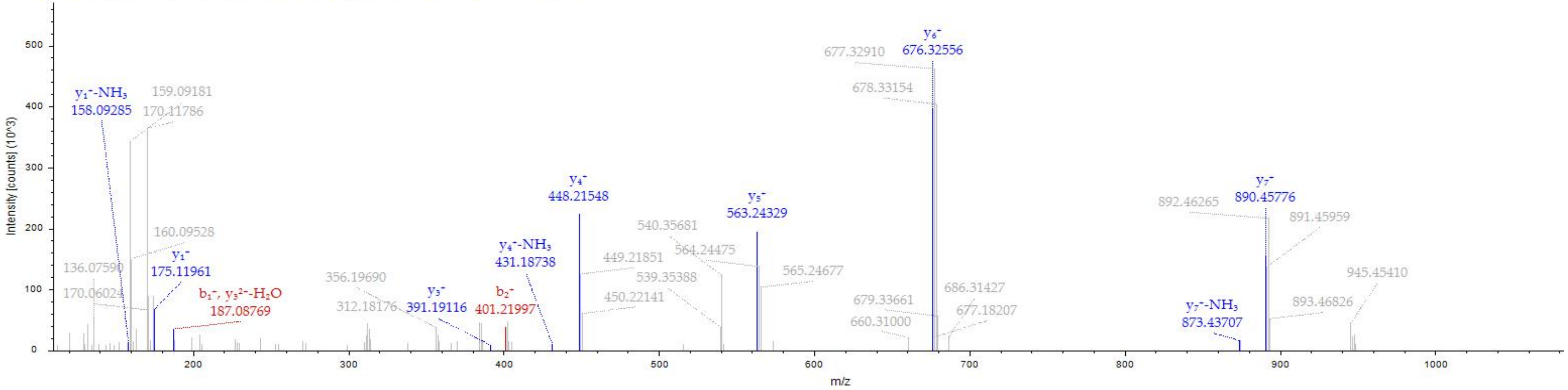

Sequence: EQMAKDK, K5-Kbhb (86.03680 Da)
Charge: +1, Monoisotopic m/z: 935.45202 Da (+1.75 mmu/+1.87 ppm), MH+: 935.45202 Da, RT: 29.5284 min,
Identified with: Sequest HT (v1.17); XCorr:1.31,
Fragment match tolerance used for search: 0.02 Da
Fragments used for search: $-H_2O$; y; $-NH_3$; y; b; b; $-H_2O$; b; $-NH_3$; y

Fragment Matches

Value Type: Theo. Mass [Da]

Ion Series | Neutral Losses | Precursor Ions | Internal Fragments

#1	b^+	Seq.	y^+	#2
1	130.04987	E		7
2	258.10845	Q	806.40769	6
3	389.14893	M	678.34911	5
4	460.18605	A	547.30862	4
5	674.31781	K-Kbhb	476.27151	3
6	789.34475	D	262.13975	2
7		K	147.11280	1

VH+CH:
EVKLEESGPGLVAPSQSLSITCTVSGFSLFSYAVHWVRQPPGKGLEWLGVIWAGGSTDYNSALMSRLSISKDNS**K**SQVFLKMNSLQTDDTAMYYCASIHYYGYWH
FDVWGAGTTVTVSSAKTTPPSVYPLAPGSAAQTNSMVTLGCLVKGYFPEPVTVTWNSGSLSSGVHTFPAVLQSDLYTLSSSVTVPSSTWPSETVTCNVAHPASSTK
VDKKIVPRDCGCKPCICTVPEVSSVFIFPPKPKDVLTITLTPKVTCVVVDISKDDPEVQFSWFVDDVEVHTAQTQPREEQFNSTFRSVSELPIMHQDWLNGKEFKCRV
NSAAFPAPIEKTISKTKGRPKAPQVYTIPPPKEQMAKDKVSLTCMITDFFPEDITVEWQWNGQPAENYKNTQPIMDTDGSYFVYSKLNVQKSNWEAGNTFTCSVLH
EGLHNHHTEKSLSHSPGK

VH+CH: Variable region of heavy chain + Constant region of heavy chain

Fragment Spectrum

MS250398-AB+-solution.raw #21933 RT: 29.5284 min
FTMS, 935.4504@hcd30.00, z=+1, Mono m/z=935.45202 Da, MH+=935.45202 Da, Match Tol.=0.02 Da

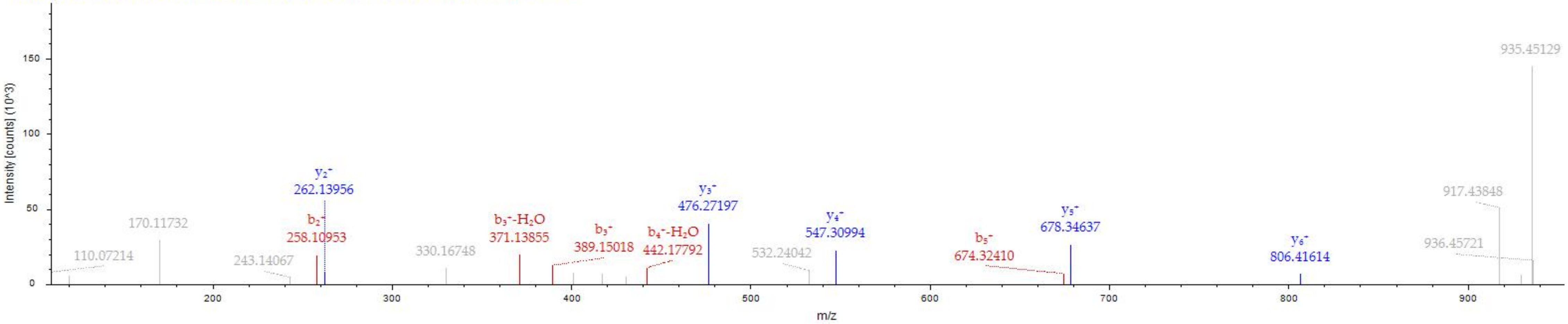

Sequence: LSISKDNSK, K5-Kbhb (86.03680 Da)
Charge: +2, Monoisotopic m/z: 539.29436 Da (+1.4 mmu/+2.59 ppm), MH+: 1077.58145 Da, RT: 36.8165 min,
Identified with: Sequest HT (v1.17); XCorr:2.46,
Fragment match tolerance used for search: 0.02 Da
Fragments used for search: -H_2O; y; -NH_3; y; b; b; -H_2O; b; -NH_3; y

Fragment Matches

Value Type: Theo. Mass [Da]

Ion Series | Neutral Losses | Precursor Ions | Internal Fragments

#1	b⁺	b²⁺	Seq.	y⁺	y²⁺	#2
1	114.09134	57.54931	L			9
2	201.12337	101.06532	S	964.49459	482.75093	8
3	314.20743	157.60735	I	877.46256	439.23492	7
4	401.23946	201.12337	S	764.37849	382.69289	6
5	615.37122	308.18925	K-Kbhb	677.34647	339.17687	5
6	730.39817	365.70272	D	463.21470	232.11099	4
7	844.44109	422.72419	N	348.18776	174.59752	3
8	931.47312	466.24020	S	234.14483	117.57605	2
9			K	147.11280	74.06004	1

VH+CH:
EVKLEESGPGLVAPSQSLSITCTVSGFSLFSYAVHWVRQPPGKGLEWLGVIWAGGSTDYNSALMSRLSISKDNSKSQVFLKMNSLQTDDTAMYYCASIHYYGYWH
FDVWGAGTTVTVSSAKTTPPSVYPLAPGSAAQTNSMVTLGCLVKGYFPEPVTVTWNSGSLSSGVHTFPAVLQSDLYTLSSSVTVPSSTWPSETVTCNVAHPASSTK
VDKKIVPRDCGCKPCICTVPEVSSVFIFPPKPKDVLTITLTPKVTCVVVDISKDDPEVQFSWFVDDVEVHTAQTQPREEQFNSTFRSVSELPIMHQDWLNGKEFKCRV
NSAAFPAPIEKTISKTKGRPKAPQVYTIPPPKEQMAKDKVSLTCMITDFFPEDITVEWQWNGQPAENYKNTQPIMDTDGSYFVYSKLNVQKSNWEAGNTFTCSVLH
EGLHNHHTEKSLSHSPGK

VH+CH: Variable region of heavy chain + Constant region of heavy chain

Fragment Spectrum

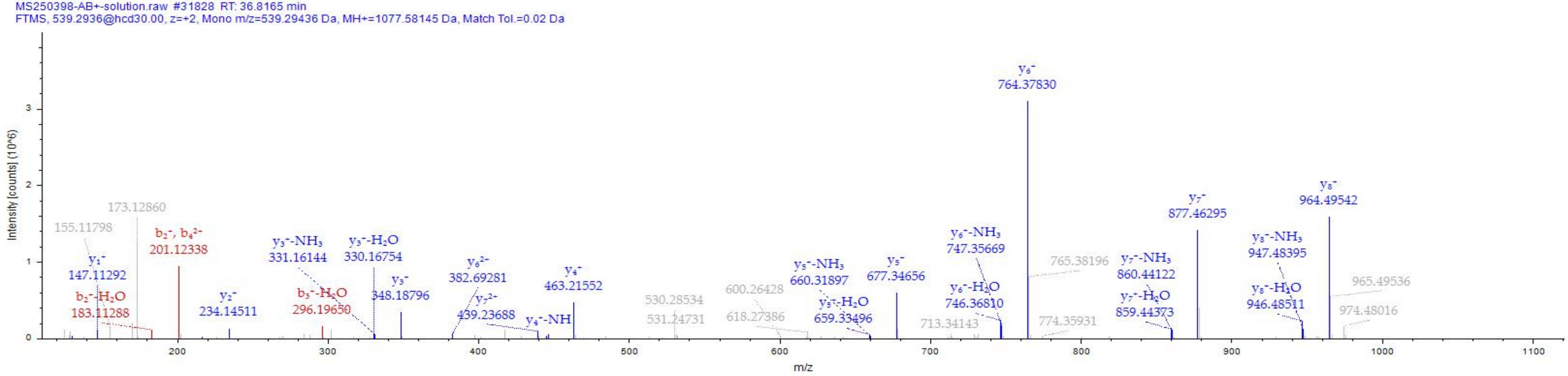

Supplement: Supplementary file 1 [file DataSheet1.pdf]
