# Supplementary figures and images for: Enhancing the structural stability of P29-targeted monoclonal antibodies via β-hydroxybutyrylation modification improves their therapeutic performance in alveolar echinococcosis
Source: Front Cell Infect Microbiol. 2026 Jan 5;15:1716047. doi: 10.3389/fcimb.2025.1716047 (PMC12812953; doi:10.3389/fcimb.2025.1716047)

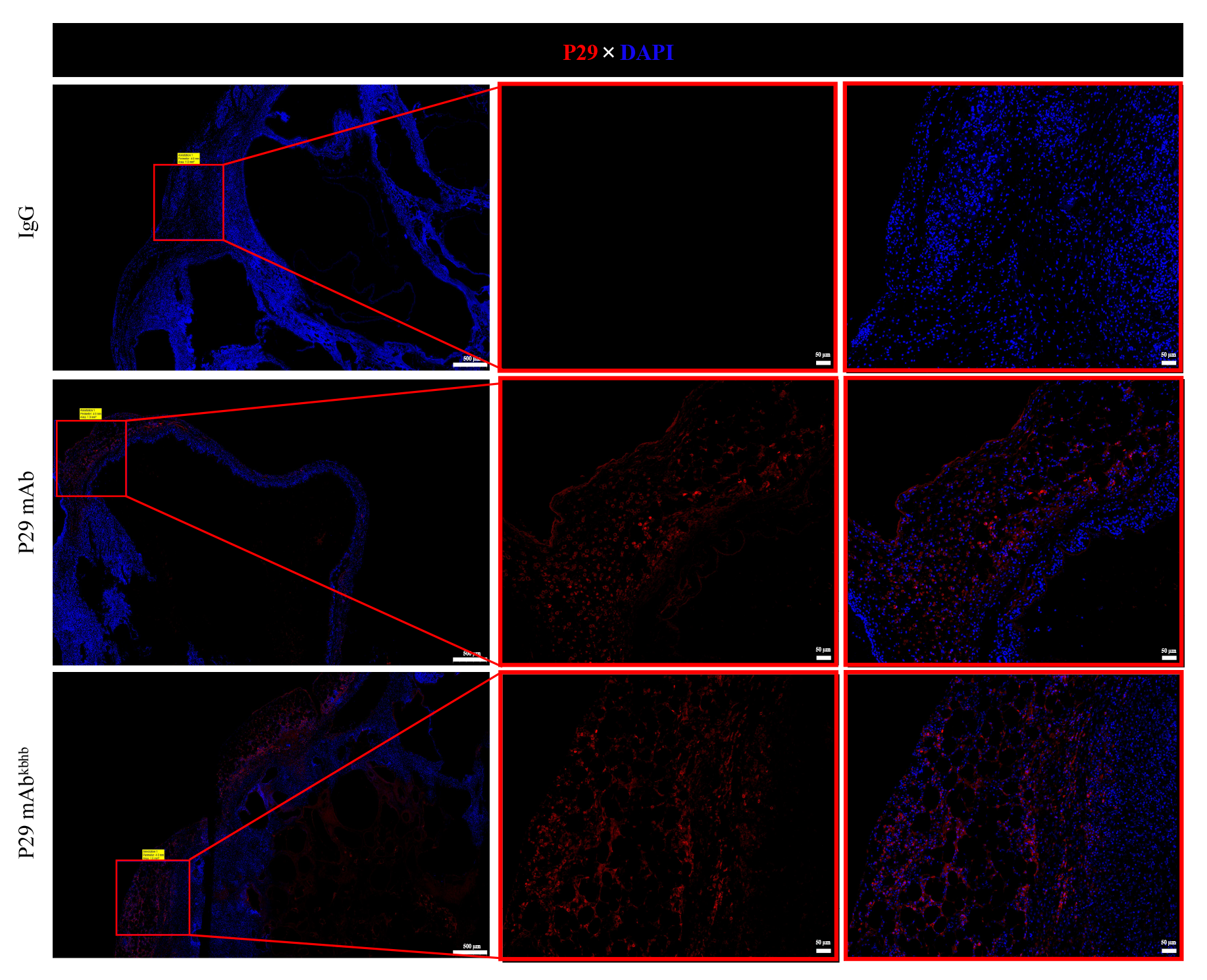

Supplement: Supplementary Figure S1 — Antigen-binding characterization of P29 mAbkbhb in alveolar hydatid cyst. Alveolar hydatid cyst tissue sections were stained with P29 mAbkbhb (red) to detect P29 antigen distribution. Nuclei were counterstained with DAPI (blue). For each treatment group, low-magnification views (left panels; scale bars = 500 μm) display the overall tissue architecture and antigen distribution patterns, with boxed regions indicating areas selected for high-magnification imaging (right panels; scale bars = 50 μm). [file Image1.tif]

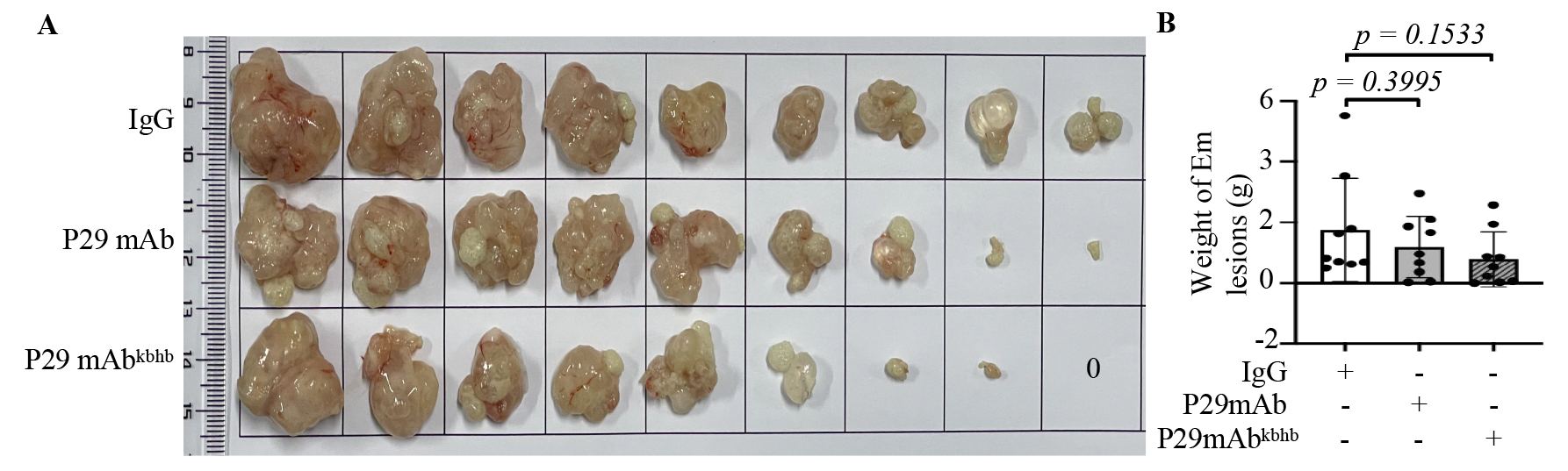

Supplement: Supplementary Figure S2 — Gross morphology and wet weight of alveolar hydatid cysts. (A) Representative images of alveolar hydatid cysts isolated from AE model mice following 8-week treatment with IgG (n=9), P29 mAb (n=9), or P29 mAbkbhb (n=9). (B) Quantitative analysis of cyst wet weight. Data are presented as mean ± SEM. Statistical significance was determined by two-tailed Student's t-test, with exact p-values indicated directly on the figure. [file Image2.tif]

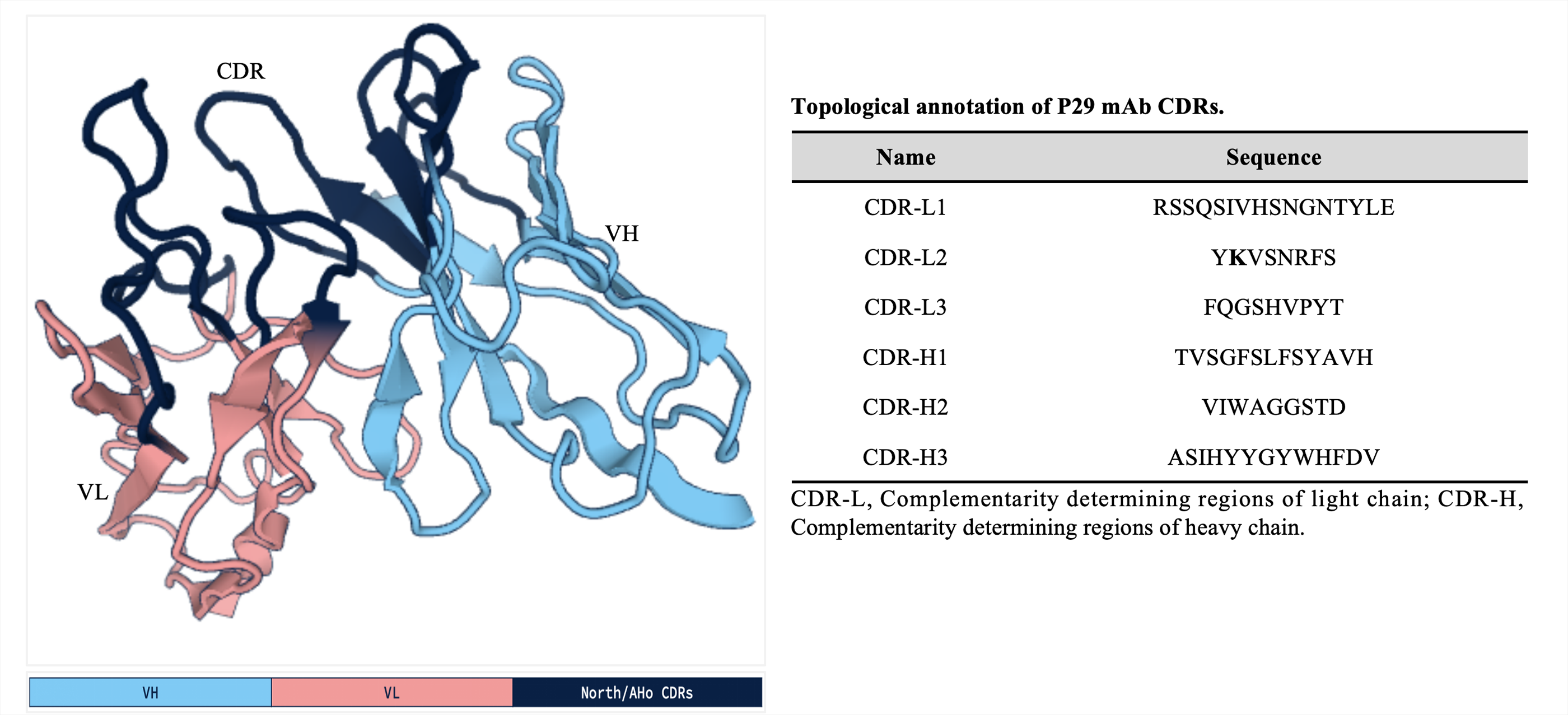

Supplement: Supplementary Figure S3 — Computational prediction of P29 mAb complementarity-determining regions (CDRs). (A) Structural model of P29 mAb variable domains. Predicted using SAbPred server with North/AHo numbering scheme, Light pink represent Variable light chain (VL), Steel blue represent Variable heavy chain (VH), Midnight blue represent Complementarity-determining regions (CDRs). [file Image3.tif]
